# Anaplastic Thyroid Carcinoma: A ceRNA Analysis Pointed to a Crosstalk between *SOX2*, *TP53*, and microRNA Biogenesis

**DOI:** 10.1155/2015/439370

**Published:** 2015-01-29

**Authors:** Walter Arancio, Valeria Carina, Giuseppe Pizzolanti, Laura Tomasello, Maria Pitrone, Concetta Baiamonte, Marco Calogero Amato, Carla Giordano

**Affiliations:** ^1^Section of Cardio-Respiratory and Endocrine-Metabolic Diseases, Biomedical Department of Internal and Specialist Medicine (Di.Bi.M.I.S.), University of Palermo, Piazza delle Cliniche 2, 90127 Palermo, Italy; ^2^Istituto Ortopedico Rizzoli (IOR), Section of Biology and Genetics, Department of Pathobiology and Medical and Forensic Biotechnology (Di.Bi.Me.F.), University of Palermo, Via Divisi 83, 90100 Palermo, Italy

## Abstract

It has been suggested that cancer stem cells (CSC) may play a central role in oncogenesis, especially in undifferentiated tumours. Anaplastic thyroid carcinoma (ATC) has characteristics suggestive of a tumour enriched in CSC. Previous studies suggested that the stem cell factor *SOX2* has a preeminent hierarchical role in determining the characteristics of stem cells in SW1736 ATC cell line. In detail, silencing SOX2 in SW1736 is able to suppress the expression of the stem markers analysed, strongly sensitizing the line to treatment with chemotherapeutic agents. Therefore, in order to further investigate the role of SOX2 in ATC, a competing endogenous RNA (ceRNA) analysis was conducted in order to isolate new functional partners of SOX2. Among the interactors, of particular interest are genes involved in the biogenesis of miRNAs (*DICER1, RNASEN,* and *EIF2C2*), in the control cell cycle (*TP53, CCND1*), and in mitochondrial activity (*COX8A*). The data suggest that stemness, microRNA biogenesis and functions, p53 regulatory network, cyclin D1, and cell cycle control, together with mitochondrial activity, might be coregulated.

## 1. Introduction

Anaplastic thyroid carcinoma (ATC) is a rare endocrine tumour. Its morphological features resemble undifferentiated neoplasm. Due to severe metastasis development and to the rapid fatal course, surgery is rarely performed. Radiotherapy and chemotherapy are also not very effective. It has been suggested that those standard therapies are ineffective because they are not able to efficiently target a subpopulation of ATC cells, called the cancer-initiating cells or cancer stem cells (CSCs). It has been proposed that CSCs possess stem-cell-like features, are at the core of the development of many tumours, especially undifferentiated ones like ATC, are responsible for the recurrence of the tumour and metastasis formation, and usually are very resistant to classical therapies.

Despite many controversies regarding the cancer stem cell model, it has the potential to drive the discovery of innovative treatments that may eradicate the very chemoresistant core of cancer [1]. In this connection, the CSC model is the sum of many hypotheses that have arisen to explain the most vexing aspects of cancer: metastasis, relapse, and therapeutic resistance [2]. In this perspective, CSC research holds out promise for improved treatment outcomes, in particular, as regards overcoming resistance to chemotherapy on solid tumours [1].

The most accepted CSC model makes use of a new paradigm of cellular differentiation, in which cancer cells can dedifferentiate toward more primitive, stem-like phenotypes [2]. The dedifferentiation seems to be highly heterogeneous, giving an explanation to the observed discontinuous behaviour of many cancers [2]. Alternatively, CSCs might arise from transformed stem cells in the stem niche [1, 2].

Similar to normal stem cells, CSCs have the ability both to self-renew and to give rise to differentiated tumour cells, are responsible for the organization of a tumour mass [3], and are tumorigenic when transplanted into an animal host [4]. CSCs have been identified in a wide range of human tumours [3]. At the molecular level, CSCs are usually enriched in cell surface markers such as CD44, CD24, and CD133, while Wnt/*β*-catenin, Notch, and Hedgehog signalling pathways seem to have key roles in CSC properties [4]. Specific microRNA signatures have been identified in many CSCs [4] that seem to play a role in the epithelial-mesenchymal transition [4].

Regarding ATC, it has been hypothesized that the tumour initiates from transformed thyroid stem cells, rather than from differentiated thyrocytes undergoing a conventional multistep carcinogenesis model [5–7].

The rarity and rapid fatal nature of ATC has led to limited* ex vivo* studies. Here we describe an* in vitro* study on a well-validated ATC cell line: SW1736. The SW1736 cell line is characterized by a high percentage of population with stem cell-like properties and high expression of several stem markers (SOX2, OCT4, NANOG, C-MYC, SSEA4, and the ABCG2 transporter) [8]. Interestingly,* SOX2* silencing downregulates* in trans* the expression of other stem cell markers and sensitizes ATC cells to treatment with classical chemotherapeutics such as cisplatin and doxorubicin [8]. This suggests that the stem cell factor* SOX2* could have a preeminent hierarchical role in determining the characteristics of stem cells in SW1736 ATC cell line.

Therefore, in order to further investigate the role of* SOX2* in ATC, a bioinformatic analysis of the functional network of* SOX2* was performed. In detail, a competing endogenous RNA (ceRNA) analysis was conducted. This kind of analysis is able to predict genes functionally correlated with the* bait* gene rather than physically associated with it [9, 10]. The ceRNA hypothesis is based on the rationale that RNA molecules can regulate one another via microRNAs [9, 10]. ceRNAs are RNAs that share miRNA recognition elements, thereby regulating each other by influencing the available level of miRNA [9, 10]. In the past, ceRNA analysis made it possible to isolate several genes and functional networks related to cancer development, ageing, and homeostasis [11–19].

## 2. Materials and Methods

### 2.1. MirWALK Analysis

miRWalk is a comprehensive database that provides information on miRNA from humans, mice, and rats on their predicted as well as validated binding sites on their target genes. The validated targets module [20] hosts experimentally verified miRNA interaction with associated genes.

Using the miRWalk [20] data and embedded tools, we collected the microRNAs that have been reported in the literature to regulate the main transcript from the SOX2 locus (Table 1).

This set of miRNAs was inserted into the miRWalk analysis tool [20] to collect any human mRNA that has been reported to be regulated by them. Then the genes collected were organized in a hierarchical order for the number of validated microRNA hits (Table 2). The more microRNAs are shared between the bait* SOX2* gene and the candidate genes, the higher the possibility that the candidate gene transcripts can act as* SOX2* ceRNAs. All analyses were updated to December 15, 2013.

### 2.2. GeneMANIA Analysis

**Figure 1:**
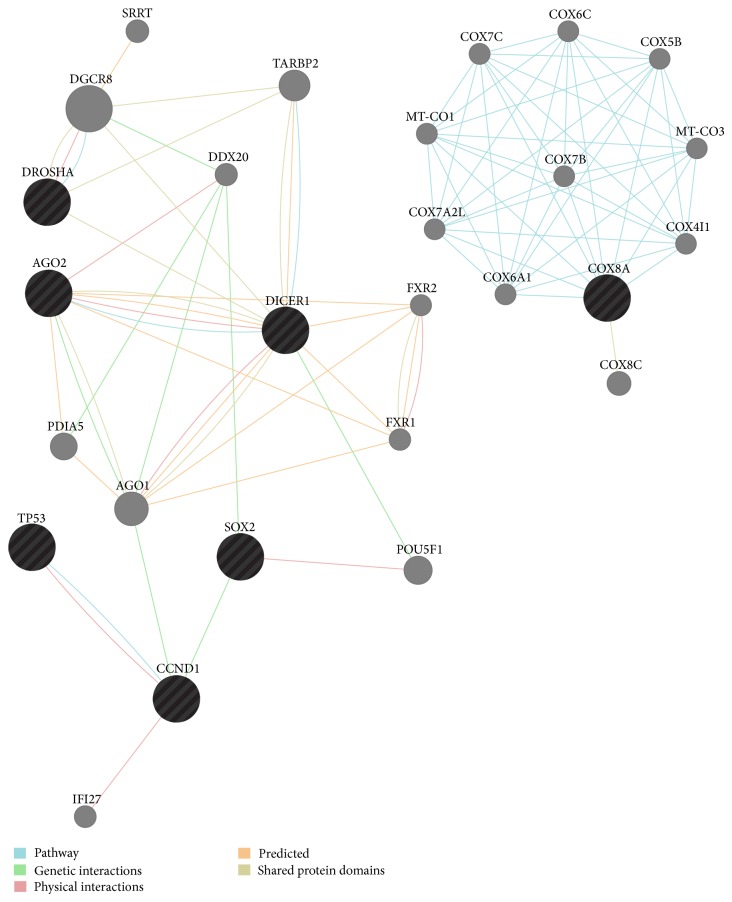
SOX2 ceRNA network by GeneMANIA.

Arbitrarily, the top 6 genes together with* SOX2* were analysed using the GeneMANIA [21] tool that helps to predict the functions of a set of genes and to predict in which gene ontology (GO) functions the set of genes might be involved (Figure 1) (Table 3). The GO functions reported are the ones with a false discovery rate (FDR) < 0.1. All analyses were updated to December 15, 2013.

### 2.3. Cell Cultures

The SW1736, 8505C, C643, FRO, BCPAP, TPC-1, and WRO cell lines were cultured in Dulbecco's modified Eagle's medium high glucose medium supplemented with 10% fetal bovine serum and 5% glutamine. Cultures were maintained in 5% carbon dioxide at 37°C in a humidified incubator.

### 2.4. Small Interfering RNA (siRNA) Transfection

siRNA transfection in SW1736 cells was performed using INTERFERin transfection agent (Polyplus-Transfection, Illkirch, France), according to the manufacturer's instructions. Briefly, the transfection agent and the siRNA complex were added to the cells and incubated for 72 hours for RNA extraction and analysis. The final concentration of* SOX2* siRNA was 100 nM. Each assay was performed in triplicate in at least three independent experiments.* SOX2* was silenced using Stealth SiRNA SOX2 HSS144045 (Invitrogen, Milan, Italy). siCONTROL Stealth siRNA Negative Control was used as a control (Invitrogen, Milan).

### 2.5. SOX2 Coding Sequence Vector and Transient Transfection

The vector used was taken from Addgen (http://www.addgene.org/) (Plasmid 26817): pcDNA3.3_SOX2; and transformation into SW1736 cells was performed using Xfect transfection agent (Clontech Laboratories, Inc. A Takara Bio Company) according to the manufacturer's instructions. The transfection agent and plasmid were added to the cells and incubated for 72 hours for RNA extraction and analysis.

### 2.6. *SOX2* 3′ Untranslated Region (3′UTR) Vector and Transfection

The vector was synthesized in service by Eurofins genomics (https://www.eurofinsgenomics.eu) using a pcDNA 3.1 backbone and a chemically synthesized 3′UTR (as reported in http://mybioinfo.info/exon_display.php?tax_id=9606&gene_id=GeneID: 6657) (Table 4).

### 2.7. Reverse-Transcription PCR and Real-Time Quantitative PCR

Total RNA was extracted from cells using the RNeasy Mini Kit (Qiagen, Milan, Italy), including a digestion step with DNase I. RNA quantity and quality were assessed using the Nanodrop 2000 (Thermo Scientific, Wilmington, USA). The RNA extracted was reverse-transcribed with Random Hexamers (Applied Biosystems, Darmstadt, Germany) and Improm II Reverse Transcriptase (Promega Italia, Milan, Italy), according to the manufacturer's protocol. Primer pair sequences are reported in Table 5.

The reactions were performed as follows: 5′ at 94°C, 30 cycles (30′′ at 94°C, 30′′ at 55°C, 30′′ at 72°C), 5′ at 72°C, and stocked at 4°C. The only exception was the amplification of the SOX2 3′UTR, for which the following was done: 5′ at 94°C, 30 cycles (30′′ at 94°C, 30′′ at 55°C, 90′′ at 72°C), 5′ at 72°C, and stocked at 4°C. Expression was analyzed by real-time quantitative PCR (qRT-PCR) using Quantitect SYBR Green PCR kit (Qiagen, Milan, Italy). All reactions were performed using a Rotor-gene Q Instrument (Qiagen, Milan, Italy). The data were analysed using the REST software [22].

### 2.8. Pool of Normal Thyroid Tissue

A pool of RNA from normal thyroid tissue specimens was used, as described in [23].

### 2.9. Limbal Stem Cell

A pool of RNA from limbal stem cells specimens was used, as described in [24].

### 2.10. Lymphocytes

Peripheral blood samples of a healthy volunteer were collected in tubes containing ethylenediaminetetraacetic acid (EDTA, 1 mg/mL) after 8 hours' fasting. Lymphocytes were isolated by lympholyte (CEDARLANE, Burlington, Ontario, Canada), according to the manufacturer's instructions.

### 2.11. Statistical Analysis

We used the SPSS 13 software, Windows edition, for all our statistical analyses. Correlations were determined using Spearman's test (nonparametric equivalent for Pearson's test). *P* < 0.05 was considered statistically significant.

## 3. Results

Previous data [8] suggested that the stem cell factor* SOX2* possesses a preeminent hierarchical role in determining stemness characteristics in the SW1736 ATC cell line. With the final aim of investigating the role of* SOX2* in ATC, a bioinformatic ceRNA analysis [9, 10] of the functional network of* SOX2* was performed.

Using the miRWalk [20] data and embedded tools, we collected the microRNAs that have been reported in the literature to regulate the main transcript from the* SOX2* locus (Table 1).

This set of miRNAs was inserted into the miRWalk analysis tool [20] to collect any human mRNA that has been reported to be regulated by them. Then the collected genes were organized in a hierarchical order for the number of validated microRNA hits (Table 2). The more microRNAs are shared between SOX2 and the candidate genes, the stronger the putative competitive effect that is at the core of the ceRNA hypothesis. The first six top level interactors were arbitrary selected for further analyses. The top level* SOX2* interactors in this ceRNA analysis are* DICER1*,* EIF2C2,* and* RNASEN*, involved in miRNA biogenesis and functions [25]; the most studied antioncogene* TP53*, worthy of note because of its suggested role in stemness [26]; the nuclear-coded mitochondrial Cytochrome C Oxidase Subunit VIIIA* COX8A* [27]; and* CCND1*, the cyclin D coding gene [28].

Amongst the lesser interactors reported in Table 2, other genes might be worth studying in the future, especially for their involvement in oncogenesis (such as* MYC*,* BCL2*,* PTEN*,* KRAS*,* JUN*, and many others).

This study aimed to analyse whether a relationship might exist between the 6 top level interactors (*DICER1*,* EIF2C2*,* RNASEN*,* TP53*,* COX8A,* and* CCND1*) and* SOX2 *in the ATC cell line SW1736.

With this purpose in mind, the six interactors together with* SOX2* were analysed by GeneMANIA software [21] to verify whether their putative network (Figure 1) might be enriched in some GO annotations. Unsurprisingly, the analysis revealed a statistically significant enrichment of miRNA-mediated, posttranscriptional gene silencing activities (Table 3).

**Figure 2:**
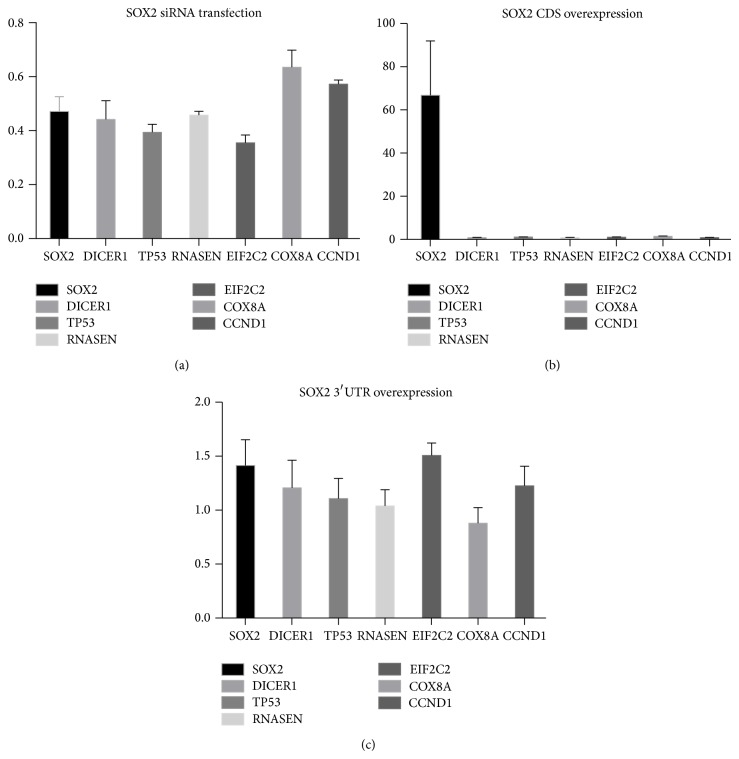
Example of SOX2 and SOX2 ceRNAs levels of transcription quantified by RT-PCR compared to controls and normalized against *β*-actin expression in SW1736 ATC cell line. Whiskers represent the standard errors. (a) Analysis of SOX2 silencing. (b) Analysis of SOX2 coding sequence overexpression. (c) Analysis of SOX2 3^'^UTR overexpression.

Then we tried to establish in the SW1736 ATC cell line whether perturbations in the transcriptional state of* SOX2* might alter* in trans* the transcriptional state of the ceRNAs identified. When we knocked down* SOX2* transcripts via specific siRNA, all the ceRNAs were coherently downregulated* in trans* in RT-PCR analyses, as expected. The effect of the downregulation varied from one ceRNA to another but was always statistically significant [22] (Figure 2(a)) (Table 6). To further investigate whether the effect could be mediated by the impaired transcriptional factor activity of the protein coded by* SOX2*, we evaluated whether the overexpression of the coding sequence of* SOX2* could have some* in trans* effects on ceRNAs. The coding sequence lacks the 3′ untranslated region (3′UTR) that mainly bears the regulation mediated by miRNAs [29]. Our data indicate that no such effect occurs (Figure 2(b)) (Table 7), so the trans effect highlighted in (Figure 2(a)) is likely to be due to the endogenous miRNA competition, as in our hypothesis, rather than a classical interaction mediated by the proteic transcriptional factor* SOX2*. Finally, we investigated whether the overexpression of* SOX2* 3′UTR might have any effects* in trans* in the SW1736 ATC cell line. The effects were very modest, if present at all, but in line with the modulation that occurs during the competing events [9, 10]. The most notable effect was the positive correlation, as expected, with the expression of* EIF2C2* and* SOX2* itself (Figure 2(c)) (Table 8).

The experiments previously described looked into the effects of perturbation of the expression of SOX2 on the expression of ceRNA genes in an ATC cell line. We then endeavoured to see whether any correlation might exist between the basal expression of* SOX2* and the ceRNA genes in different specimens. In detail, we analysed by RT-PCR the relative expression of* SOX2* and* SOX2* ceRNAs compared to * β-ACTIN* expression in SW1736 ATC cell line, in 8505C ATC cell line, C643 ATC cell line, FRO ATC cell line, BCPAP papillary thyroid carcinoma (PTC) cell line, TPC-1 PTC cell line, WRO follicular thyroid carcinoma, and a pool of normal thyroid tissues present in the laboratory from previous experiments [23], a pool of limbal stem cells [24], and isolated lymphocytes from a male donor of 36 years old (see Supplementary Table 1 in Supplementary Material available online at http://dx.doi.org/10.1155/2014/439370). Interestingly, this analysis suggested a correlation in the basal expression of* DICER1*,* RNASEN,* and* EIF2C2* (Table 9), as can be expected of genes whose functions are strictly coregulated in the biogenesis and function of microRNA, but surprisingly their basal expression seemed also to be somehow related to the basal expression of* TP53* (Table 9), suggesting interesting scenarios that will be discussed shortly.

## 4. Discussion

The ceRNA bioinformatics analysis pointed to a list of genes that could be functionally coregulated with the stem transcriptional factor* SOX2* by a crosstalk mediated by several miRNAs. In our analysis, we used interactions reported in the literature instead of bioinformatically predicted ones as done in the past [17–19]. This approach makes it possible to harvest more solid and reliable data, though it is easier to collect genes that have been previously analysed.

Our experiments were pursued in an ATC cell line that has previously been demonstrated to constitutively express* SOX2* that functionally possesses a preeminent hierarchical role on many other stem cell factors [8], suggesting a leading role in the maintenance of the stemness feature in this cell line. ATC represents a very good candidate for a cancer highly enriched in CSCs, which probably are at the core of its unfavourable outcome [1–7]. For these reasons, it is both important to understand the regulatory network that underlies the functions of* SOX2* in ATC, and at the same time an ATC cell line is a very good candidate for studying the* SOX2* network.

Looking at the cross-regulation between* SOX2* and the most probable ceRNAs that we isolated, many if not all the ceRNAs analysed seem to be responsive to alterations in the transcriptional state of* SOX2* transcripts, independently of the coded protein, suggesting a regulatory network strictly based on noncoding-RNAs (ncRNAs). The most striking evidence is the effect of siRNA-mediated silencing on* SOX2*, where all the ceRNAs are accordingly downregulated (Figure 1(a)) (Table 6). By contrast, the overexpression of the* SOX2* CDS alone seems to have almost no effect at all (Figure 1(b)) (Table 7). In contrast, the overexpression of the 3′UTR of the* SOX2* transcripts seems to have an upregulation effect* in trans*, even if not to a great degree (Figure 1(c)) (Table 8). The 3′UTR of transcripts is the portion of messengers that is likely to bear the vast majority of regulation mediated by microRNAs [30]. The data reported here are consistent with our hypothesis, so it is reasonable to point to the ceRNAs isolated as potential functional interactors with* SOX2*, at least in the SW1736 ATC cell line.

The interactors isolated pointed to a central role of microRNA biogenesis and functions in* SOX2* activities (Table 3) and hence in stemness, as other authors have recently suggested [29]. Here we report that probably the transcription of* SOX2* stem factors and of Dicer (*DICER1*), Ago2 (*EIF2C2*), and Drosha (*RNASEN*) is coregulated by a microRNA network. In detail, Drosha is a RNA-specific endoribonuclease that is involved in the initial nuclear step of microRNA biogenesis. Dicer is a cytoplasmic endoribonuclease that plays a central role in the production of short interfering RNAs (siRNA) and mature microRNAs. SiRNAs and microRNAs serve as a guide to directing the RNA-induced silencing complex (RISC) to complementary RNAs to degrade them or prevent their translation. Ago2 is the essential proteic core of the RISC complex. Overall, the miRNA pathway is a means to specifically regulate the expression of target genes that seem to directly and indirectly affect tumorigenesis [31].

The* SOX2* ceRNA* TP53* gene codes for p53, one of the most studied genes in relation to cancer development [32]. It is also often mutated in ATC towards a nonfunctional form [33]. Nevertheless, even a mutated form, if transcribed, can still exert its regulatory functions via its transcript (e.g., its 3′UTR). In this perspective, homozygous deletion of the locus or full silencing of the gene perturbs the network differently from a null mutation, which still permits transcription from the locus. In the authors' opinion, this distinction is often not taken into account. It is interesting to note that some authors have suggested a role of p53 in homoeostasis of the stem niche [26] and in microRNA biogenesis [34], setting it at a crossroads between cancer, stemness, and microRNA biogenesis and functions. Our data are in support of this interpretation, all the more so because the basal transcription of* TP53* seems to be correlated with the basal transcription of* DICER1*,* EIF2C2,* and* RNASEN *in the specimens that we analysed, many of them from ATC and other thyroid cell lines (Table 9).

The* SOX2* ceRNA* CCND1* codes for cyclin D1, the regulatory subunit that promotes G1/S cell-cycle progression and is involved in oncogenesis. It has been reported that cyclin D1 induces Dicer expression* in vitro* and* in vivo* and vice versa and their expression significantly correlates each other (at least in some subtypes of human breast cancer). It has been suggested that cyclin D1 induction of Dicer coordinates microRNA biogenesis [28, 35]. Our data are in line with the previous results and add a new level of possible crosstalk between* DICER1* and* CCND1*, suggesting novel actors in the network previously isolated, such as* SOX2* or* TP53*. It is likely that cross-regulation between cyclin D1 and Dicer might occur in other cancers, especially in ATC, which are enriched in* SOX2* producing cells [8], which we suggest is part of the network.

The role of* COX8A* is more difficult to appraise. The protein encoded by this gene is the terminal enzyme of the respiratory chain that leads to the production of the electrochemical gradient across the inner mitochondrial membrane. Recent discoveries suggest central roles of mitochondria in the maintenance of pluripotency, differentiation, reprogramming, and ageing [36]. Our data suggest possible crosstalk between a crucial nuclear coded mitochondrial factor and cell fate determinants such as* SOX2* and* TP53*.

## 5. Conclusions

The SW1736 ATC cell line was used to investigate functional* SOX2* interactors isolated by a novel bioinformatics analysis. Because* SOX2* seems to have a central role in the maintenance of stem features in the SW1736 ATC cell line, the interactors are likely to play a role in stemness regulation.

The analysis pointed to* DICER1, EIF2C2, RNASEN, TP53, COX8A,* and* CCND1* genes, suggesting that stemness, microRNA biogenesis and functions, p53 regulatory network, cyclin D1, and cell cycle control, together with mitochondrial activity, might be coregulated as a whole in their functions. Our data and previous data from the literature indicate that those functions are strictly interlinked and that deregulation of them might lead to cancer transformation, especially in cancers such as ATC that possess an undifferentiated nature suggestive of cancer stem cell enrichment.

## Supplementary Material

In order to analyse the basal expression of SOX2 and the ceRNA genes in different specimens we analysed by RT-PCR the relative expression of SOX2 and SOX2 ceRNAs compared to β-ACTIN expression in: SW1736 ATC cell line, 8505C ATC cell line, C643 ATC cell line, FRO ATC cell line, BCPAP papillary thyroid carcinoma (PTC) cell line, TPC- 1 PTC cell line, WRO follicular thyroid carcinoma, a pool of normal thyroid tissues, a pool of limbal stem cells, and isolated lymphocytes from a male donor of 36 years old. These data have been used to thest theri correlation as reported in Table 9.

## Figures and Tables

**Table 1:** microRNAs that have been reported in the literature to regulate the main transcript from the SOX2 locus.

hsa-let-7a	hsa-miR-125b-2^*^	hsa-miR-1914^*^	hsa-miR-30a^*^
hsa-let-7a^*^	hsa-miR-126	hsa-miR-1915	hsa-miR-30b
hsa-let-7b	hsa-miR-126^*^	hsa-miR-1915^*^	hsa-miR-30b^*^
hsa-let-7b^*^	hsa-miR-134	hsa-miR-200c	hsa-miR-30c
hsa-let-7c	hsa-miR-137	hsa-miR-200c^*^	hsa-miR-30c-1^*^
hsa-let-7c^*^	hsa-miR-142-3p	hsa-miR-203	hsa-miR-30c-2^*^
hsa-let-7d	hsa-miR-143	hsa-miR-204	hsa-miR-30d
hsa-let-7d^*^	hsa-miR-143^*^	hsa-miR-205	hsa-miR-30d^*^
hsa-let-7e	hsa-miR-145	hsa-miR-206	hsa-miR-30e
hsa-let-7e^*^	hsa-miR-145^*^	hsa-miR-21	hsa-miR-30e^*^
hsa-let-7f	hsa-miR-155	hsa-miR-21^*^	hsa-miR-452
hsa-let-7f-1^*^	hsa-miR-155^*^	hsa-miR-223	hsa-miR-452^*^
hsa-let-7f-2^*^	hsa-miR-17	hsa-miR-223^*^	hsa-miR-9
hsa-let-7g	hsa-miR-17^*^	hsa-miR-296-3p	hsa-miR-9^*^
hsa-let-7g^*^	hsa-miR-183	hsa-miR-296-5p	hsa-miR-92a
hsa-let-7i	hsa-miR-183^*^	hsa-miR-302a	hsa-miR-93
hsa-let-7i^*^	hsa-miR-1908	hsa-miR-302a^*^	hsa-miR-93^*^
hsa-miR-100	hsa-miR-1909	hsa-miR-302b	
hsa-miR-100^*^	hsa-miR-1909^*^	hsa-miR-302b^*^	
hsa-miR-106b	hsa-miR-1910	hsa-miR-302c	
hsa-miR-106b^*^	hsa-miR-1911	hsa-miR-302c^*^	
hsa-miR-125a-3p	hsa-miR-1911^*^	hsa-miR-302d	
hsa-miR-125a-5p	hsa-miR-1912	hsa-miR-302d^*^	
hsa-miR-125b	hsa-miR-1913	hsa-miR-302f	
hsa-miR-125b-1^*^	hsa-miR-1914	hsa-miR-30a	

**Table 2:** ceRNA organized in hierarchical order for the number of validated microRNA hits.

Gene	Hits	Gene	Hits
DICER1	35	SLC27A4	10
TP53	26	RUNX1	10
RNASEN	22	RRBP1	10
EIF2C2	22	PAK3	10
COX8A	22	NFKB1	10
CCND1	22	LIN28	10
MYC	20	KLF4	10
CDKN1A	20	FRAP1	10
BCL2	20	EIF2C1	10
AKT1	19	CREB1	10
PTEN	18	CDK6	10
CDKN2A	18	APC	10
VEGFA	16	TWIST1	9
EGFR	16	SYNE1	9
TGFB1	14	SIRT1	9
KRAS	14	PRDM1	9
JUN	14	MCL1	9
HMGA2	14	HMOX1	9
ERBB2	14	DNMT1	9
ZEB1	13	DDX20	9
TLR4	13	CKAP4	9
SSSCA1	13	CDKN1C	9
MET	13	BRCA1	9
TNF	12	ZNF828	8
SOCS1	12	TP63	8
PIK3CA	12	TIMM8A	8
ESR1	12	STMN1	8
DGCR8	12	SCPEP1	8
CEBPB	12	ROS1	8
CD4	12	PSAT1	8
TGFBR2	11	PDCD4	8
STAT3	11	MYCN	8
PROM1	11	MAPK3	8
NPC1	11	JAK2	8
IL6	11	IL1B	8
EPHB2	11	IFNG	8
E2F3	11	IFNA1	8
E2F1	11	HMGA1	8
CDKN1B	11	GEMIN4	8
		CTNNB1	8
		CD19	8
		BCL2L11	8
		BAX	8

**Table 3:** Gene ontology of SOX2 ceRNA network by GeneMANIA.

Function	False discovery rate	Coverage
Query genes	n/a	7/7
Gene silencing by RNA	3.14*e* − 15	9/33
Gene silencing	5.01*e* − 13	9/59
Gene silencing by miRNA	1.3*e* − 11	7/25
Posttranscriptional gene silencing by RNA	1.92*e* − 11	7/28
Posttranscriptional gene silencing	1.92*e* − 11	7/28
Regulation of gene expression, epigenetic	5.32*e* − 9	8/110
Production of miRNAs involved in gene silencing by miRNA	1.24*e* − 8	5/13
dsRNA fragmentation	1.49*e* − 8	5/14
Production of small RNA involved in gene silencing by RNA	1.49*e* − 8	5/14
Cellular response to dsRNA	7.77*e* − 8	5/19
Response to dsRNA	1.16*e* − 7	5/21
Respiratory electron transport chain	1.16*e* − 7	7/102
Electron transport chain	1.16*e* − 7	7/103
Cellular respiration	7.68*e* − 7	7/136
Mitochondrial membrane	3.2*e* − 6	8/274
ncRNA metabolic process	5.44*e* − 6	7/185
Mitochondrial envelope	5.44*e* − 6	8/297
Mitochondrial inner membrane	7.4*e* − 6	7/195
Organelle inner membrane	1.1*e* − 5	7/208
Posttranscriptional regulation of gene expression	4.71*e* − 5	7/259
Energy derivation by oxidation of organic compounds	7.46*e* − 5	7/279
Cellular response to organic cyclic compound	2.23*e* − 4	5/101
ncRNA processing	2.59*e* − 4	5/105
Endonuclease activity, active with either ribo- or deoxyribonucleic acids and producing 5′-phosphomonoesters	6.97*e* − 4	3/14
Endoribonuclease activity	1.25*e* − 3	3/17
Response to organic cyclic compound	3.56*e* − 3	5/183
Stem cell maintenance	1.09*e* − 2	3/35
Stem cell development	1.25*e* − 2	3/37
Ribonuclease activity	1.97*e* − 2	3/44
Endonuclease activity	1.97*e* − 2	3/44
Stem cell differentiation	4.17*e* − 2	3/57
Somatic stem cell maintenance	5.63*e* − 2	2/11
Germplasm	5.63*e* − 2	2/11
P granule	5.63*e* − 2	2/11
Pole plasm	5.63*e* − 2	2/11
Ribonucleoprotein granule	6.92*e* − 2	3/71
Endodermal cell fate commitment	7.54*e* − 2	2/13

**Table 4:** *SOX2* 3′ untranslated region (3′UTR).

5′GGGCCGGACAGCGAACTGGAGGGGGGAGAAATTTTCAAAGAAAAACGAGGGAAATGGGAGGGGTGCAAAA
GAGGAGAGTAAGAAACAGCATGGAGAAAACCCGGTACGCTCAAAAAGAAAAAGGAAAAAAAAAAATCCCATC
ACCCACAGCAAATGACAGCTGCAAAAGAGAACACCAATCCCATCCACACTCACGCAAAAACCGCGATGCCGAC
AAGAAAACTTTTATGAGAGAGATCCTGGACTTCTTTTTGGGGGACTATTTTTGTACAGAGAAAACCTGGGGA
GGGTGGGGAGGGCGGGGGAATGGACCTTGTATAGATCTGGAGGAAAGAAAGCTACGAAAAACTTTTTAAAAG
TTCTAGTGGTACGGTAGGAGCTTTGCAGGAAGTTTGCAAAAGTCTTTACCAATAATATTTAGAGCTAGTCTCC
AAGCGACGAAAAAAATGTTTTAATATTTGCAAGCAACTTTTGTACAGTATTTATCGAGATAAACATGGCAAT
CAAAATGTCCATTGTTTATAAGCTGAGAATTTGCCAATATTTTTCAAGGAGAGGCTTCTTGCTGAATTTTGA
TTCTGCAGCTGAAATTTAGGACAGTTGCAAACGTGAAAAGAAGAAAATTATTCAAATTTGGACATTTTAATT
GTTTAAAAATTGTACAAAAGGAAAAAATTAGAATAAGTACTGGCGAACCATCTCTGTGGTCTTGTTTAAAAA
GGGCAAAAGTTTTAGACTGTACTAAATTTTATAACTTACTGTTAAAAGCAAAAATGGCCATGCAGGTTGACA
CCGTTGGTAATTTATAATAGCTTTTGTTCGATCCCAACTTTCCATTTTGTTCAGATAAAAAAAACCATGAAAT
TACTGTGTTTGAAATATTTTCTTATGGTTTGTAATATTTCTGTAAATTTATTGTGATATTTTAAGGTTTTCCC
CCCTTTATTTTCCGTAGTTGTATTTTAAAAGATTCGGCTCTGTATTATTTGAATCAGTCTGCCGAGAATCCAT
GTATATATTTGAACTAATATCATCCTTATAACAGGTACATTTTCAACTTAAGTTTTTACTCCATTATGCACAG
TTTGAGATAAATAAATTTTTGAAATATGGACACTGAAA3′

**Table 5:** RT-PCR primer pairs.

Gene	Forward primer 5′ > 3′	Reverse primer 5′ > 3′
*SOX2* CDS	GGAGACGGAGCTGAAGCCGC	GACGCGGTCCGGGCTGTTTT
*DICER1 *	CTTTGCAACCCCTCAGCAT	TCATGAATTGCTTCTTGTTGC
*TP53 *	ATCTACTGGGACGGAACAGC	GTGAGGCTCCCCTTTCTTG
*RNASEN *	CACCGAGATCACAGTCATGG	TGTCTTCTCCTGTCGGGACT
*EIF2C2 *	TCCACCTAGACCCGACTTTG	AACTCTCCTCGGGCACTTCT
*COX8A *	TTACCTCCTGCTTCGTGACC	CACTCTGGCCTCCTGTAGGT
*CCND1 *	ATGCCAACCTCCTCAACG	GGACCTCCTTCTGCACACAT
*SOX2* 3′UTR	CACCGGGCCGGACAGCGAACTGGAGGGGGG	TTTCAGTGTCCATATTTCAAAAATTTATTTATC
*β-ACTIN *	GGACTTCGAGCAAGAGATGG	AGCACTGTGTTGGCGTACAG

**Table 6:** Example of REST analysis on SOX2 and SOX2 ceRNAs levels of transcription quantified by RT-PCR compared to controls and normalized against *β*-actin expression in SW1736 ATC cell line after SOX2 silencing. P(H1) is the probability of the alternative hypothesis that the difference between sample and control groups is due only to chance.

Gene	Reaction efficiency	Expression	Std. error	95% C.I.	P(H1)	Result
SOX2	0.6375	**0.471**	0.416–0.538	0.384–0.580	0.000	DOWN
DICER1	0.6125	**0.442**	0.373–0.523	0.366–0.533	0.000	DOWN
TP53	0.7025	**0.394**	0.364–0.427	0.348–0.445	0.170	DOWN
RNASEN	0.73	**0.458**	0.444–0.472	0.444–0.472	0.000	DOWN
EIF2C2	0.7475	**0.355**	0.326–0.386	0.320–0.394	0.000	DOWN
COX8A	0.69	**0.635**	0.572–0.705	0.560–0.720	0.000	DOWN
CCND1	0.6875	**0.572**	0.556–0.588	0.556–0.588	0.000	DOWN

**Table 7:** Example of REST analysis on SOX2 and SOX2 ceRNAs levels of transcription quantified by RT-PCR compared to controls and normalized against *β*-actin expression in SW1736 ATC cell line after SOX2 coding sequence (CDS) overexpression. P(H1) is the probability of the alternative hypothesis that the difference between sample and control groups is due only to chance.

Gene	Reaction efficiency	Expression	Std. error	95% C.I.	P(H1)	Result
SOX2	0.63	**66.783**	41.627–98.592	37.461–120.196	0.000	UP
DICER1	0.67	**0.847**	0.750–0.981	0.677–1.034	0.131	
TP53	0.8175	**1.099**	1.000–1.198	0.953–1.261	0.136	
RNASEN	0.7475	**0.840**	0.707–1.001	0.672–1.059	0.131	
EIF2C2	0.7725	**1.015**	0.829–1.246	0.788–1.320	0.854	
COX8A	0.765	**1.430**	1.197–1.699	1.136–1.788	0.000	UP
CCND1	0.755	**0.861**	0.763–0.947	0.721–1.056	0.080	

**Table 8:** Example of REST analysis on SOX2 and SOX2 ceRNAs levels of transcription quantified by RT-PCR compared to controls and normalized against *β*-actin expression in SW1736 ATC cell line after SOX2 3′UTR overexpression. P(H1) is the probability of the alternative hypothesis that the difference between sample and control groups is due only to chance.

Gene	Reaction efficiency	Expression	Std. error	95% C.I.	P(H1)	Result
SOX2	0.655	**1.414**	1.176–1.710	1.102–1.818	0.000	UP
DICER1	0.7025	**1.207**	0.952–1.536	0.912–1.600	0.680	
TP53	0.77	**1.105**	0.918–1.332	0.881–1.386	0.661	
RNASEN	0.75	**1.039**	0.889–1.222	0.833–1.299	0.830	
EIF2C2	0.765	**1.508**	1.393–1.641	1.314–1.733	0.169	UP
COX8A	0.785	**0.879**	0.733–1.068	0.669–1.160	0.680	
CCND1	0.7425	**1.226**	1.045–1.462	0.937–1.611	0.341	
*SOX2* 3′UTR	0.64	**3.092**	2.577–3.762	2.330–4.119	0.000	UP

**Table 9:** Spearman two-tailed test correlations between basal gene expressions (as reported in Supplementary Table 1) among SW1736, 8505C, C643, FRO, BCPAP, TPC-1, WRO, normal thyroid pool, limbal stem cells, and lymphocytes.

	*SOX2 *	DICER1	TP53	RNASEN	EIF2C2	COX8A	CCND1
SOX2							
Rho	1	0.152	0.2	−0.176	0.042	0.321	−0.467
*P*		0.676	0.580	0.627	0.907	0.365	0.174
DICER1							
Rho	0.152	1	0.939	0.709	0.952	0.030	−0.067
*P*	0.676	—	<0.001	0.022	<0.001	0.934	0.855
TP53							
Rho	0.200	0.939	1	0.770	0.939	−0.115	−0.042
*P*	0.580	<0.001	—	0.009	<0.001	0.751	0.907
RNASEN							
Rho	−0.176	0.709	0.770	1	0.842	−0.382	0.261
*P*	0.627	0.022	0.009	—	0.002	0.276	0.467
EIF2C2							
Rho	0.042	0.952	0.939	0.842	1	−0.127	0.067
*P*	0.907	<0.001	<0.001	0.002	—	0.726	0.855
COX8A							
Rho	0.321	0.030	−0.115	−0.382	−0.127	1	−0.127
*P*	0.365	0.934	0.751	0.276	0.726	—	0.726
CCND1							
Rho	−0.467	−0.067	−0.042	0.261	0.067	−0.127	1
*P*	0.174	0.855	0.907	0.467	0.855	0.726	—
