# Supplementary material for: Anaplastic Thyroid Carcinoma: A ceRNA Analysis Pointed to a Crosstalk between SOX2, TP53, and microRNA Biogenesis
Source: Int J Endocrinol. 2015 Jan 29;2015:439370. doi: 10.1155/2015/439370 (PMC4326218; doi:10.1155/2015/439370)
Supplement: Supplementary file 1 — In order to analyse the basal expression of SOX2 and the ceRNA genes in different specimens we analysed by RT-PCR the relative expression of SOX2 and SOX2 ceRNAs compared to β-ACTIN expression in: SW1736 ATC cell line, 8505C ATC cell line, C643 ATC cell line, FRO ATC cell line, BCPAP papillary thyroid carcinoma (PTC) cell line, TPC- 1 PTC cell line, WRO follicular thyroid carcinoma, a pool of normal thyroid tissues, a pool of limbal stem cells, and isolated lymphocytes from a male donor of 36 years old. These data have been used to thest theri correlation as reported in Table 9. [file 439370.f1.pdf]

Sup. Tab. 1: ΔCt vs β-Actin: difference between the Cycle threshold (Ct) of the gene of interest and the Ct of β-Actin reference sequence in different specimens in RT-PCR.

**ΔCt vs β-Actin**	**SOX2**	**DICER1**	**TP53**	**RNASEN**	**EIF2C2**	**COX8A**	**CCND1**
**SW1736**	12,59±0,26	10,58±0,14	2,23±0,34	7,34±0,30	5,56±0,50	-3,31±0,37	-0,52±0,29
**8505C**	16,05±0,51	7,44±0,26	-3,82±0,06	2,48±0,01	1,42±0,74	-2,33±0,10	-2,60±0,40
**C643**	15,82±0,76	5,41±0,07	-3,74±0,40	3,16±0,07	1,02±0,06	-3,05±0,14	-2,32±0,24
**FRO**	10,03±0,16	5,18±0,84	-4,23±0,23	3,05±0,19	-1,05±0,11	-8,25±0,04	-1,49±0,15
**BCPAP**	10,97±0,06	9,34±0,13	0,75±1,01	6,75±0,54	3,33±0,35	-4,17±0,10	0,15±0,07
**TPC-1**	11,19±0,91	9,29±0,40	0,59±0,30	8,46±0,13	3,40±0,23	-5,94±0,19	1,26±0,18
**WRO**	12,42±0,34	9,41±0,26	0,81±0,27	8,31±0,33	3,47±0,04	-4,43±0,90	-2,41±0,08
**Normal Thyroid Pool**	13,52±0,63	6,91±0,96	-1,66±0,08	5,62±0,71	2,13±0,59	-9,18±0,73	-1,28±0,46
**Limbal Stem Cells**	11,55±0,58	9,69±0,08	1,53±0,54	7,77±0,93	3,95±0,44	-5,52±0,47	-4,16±0,32
**Lymphocytes**	10,84±0,36	5,89±0,58	-3,94±0,51	4,77±0,05	1,87±0,72	-2,36±0,41	0,13±0,91
